# Rapid immunochromatographic tests for the diagnosis of chronic Chagas disease in at-risk populations: A systematic review and meta-analysis

**DOI:** 10.1371/journal.pntd.0007271

**Published:** 2019-05-31

**Authors:** Andrea Angheben, Dora Buonfrate, Mario Cruciani, Yves Jackson, Julio Alonso-Padilla, Joaquim Gascon, Federico Gobbi, Giovanni Giorli, Mariella Anselmi, Zeno Bisoffi

**Affiliations:** 1 Department of Infectious – Tropical Diseases and Microbiology, IRCCS Sacro Cuore - Don Calabria Hospital, Negrar, Verona, Italy; 2 Infectious Diseases Unit, Azienda ULSS 9 Scaligera, Verona, Italy; 3 Division of Primary Care Medicine, Geneva University Hospitals, Geneva, Switzerland; 4 Institute of Global Health, Geneva University, Geneva, Switzerland; 5 ISGlobal, Hospital Clínic - Universitat de Barcelona, Barcelona, Spain; 6 Centro de Epidemiologia Comunitaria y Medicina Tropical (CECOMET), Esmeraldas, Ecuador; 7 Diagnostic and Public Health Department, University of Verona, Verona, Italy; Universidad de Buenos Aires, ARGENTINA

## Abstract

**Background:**

Despite of a high disease burden, mainly in Latin America, Chagas disease (CD) is underdiagnosed and undertreated. Rapid diagnostic tests (RDTs) might improve the access to diagnosis. The aim of this study is to review the accuracy of commercially available RDTs used in field conditions for the diagnosis of chronic CD in populations at risk, in endemic and non-endemic countries.

**Methods/Principal findings:**

We undertook a comprehensive search of the following databases: PubMed, SCOPUS, LILACS (last up-date on the 01^st^ July, 2017), without language or date limits. Non-electronic sources have been also searched. This review included clinical studies with cohort recruitment of individuals at risk of *T*. *cruzi* exposure, without age limits; adequate reference standards for the diagnosis of CD. We excluded case-control studies and those testing RDTs during acute CD. Data on test accuracies were pooled through a bivariate random-effects model. Only one index test was evaluated separately. Geographical area, commercial brand, disease prevalence, study size, and risk of bias were explored as possible source of heterogeneity. Values of sensitivity and specificity were computed to obtain summary positive/negative likelihood ratios, and summary diagnostic odds ratio. Ten studies were included on six different immunochromatographic RDTs. The pooled sensitivity and specificity of the RDTs resulted 96.6% (95% CI 91.3–98.7%) and 99.3% (95% CI 98.4–99.7%), respectively. Test accuracy was particularly good in endemic areas (98.07%/99.03% of sensitivity/specificity, respectively). One test (Stat-Pak) showed an overall sensitivity of 97% (95% CI 87.6–99.3) and specificity of 99.4% (95% CI 98.6–99.8).

**Conclusions/Significance:**

RDTs demonstrated to be sufficiently accurate to recommend their use for screening in endemic areas, even as stand-alone tests. This approach might increase the accessibility to the diagnosis. However, an additional confirmatory test in case of positive result remains a prudent approach.

## Author summary

Chagas disease (CD) is a parasitic disease that can affect seriously the health status of affected individuals. People with CD, mainly living in remote areas of Latin America, often face major barriers to the disease recognition, diagnosis and treatment. The World Health Organization recommends the combined use of two tests for diagnosis of the disease in the chronic phase, but this approach is expensive, has time-constraints, and requires well-equipped laboratories, among others. Rapid diagnostic tests (RDTs) are easy-to-use, cheaper and less time-consuming than classical techniques. Hence, their large-scale use could contribute to increase the access to diagnosis, improve treatment coverage, and reduce disease transmission. We reviewed the existing studies on the accuracy of RDTs for the diagnosis of chronic CD. The RDTs under study demonstrated sufficiently good to recommend their use for screening in endemic areas (particularly the Gran Chaco), even as stand-alone tests. On the other hand, not enough evidence has been retrieved on the use of RDTs in other settings. The use of RDTs might increase the access to the diagnosis, particularly in the Gran Chaco area of Latin America.

## Introduction

Chagas disease (CD) is a parasitic disease affecting more than 8 million people and causing 806,170 DALYs lost, annually, in the endemic countries of Latin America (LA)[1]. It is caused by the protozoan parasite *Trypanosoma cruzi*, generally transmitted by insect vectors. Following international migration, the disease has spread also to non-endemic countries, where it can be transmitted congenitally or through organ or blood donation[2]. It has been recently estimated that in Northern America (Mexico, United States and Canada) from 1.3 to 7 million people could be affected[3].

CD has been associated to poverty as it causes relevant morbidity and mortality in working-age people and predominantly affects disadvantaged populations[1]. Moreover, the transplacental transmission[4] causes abortion, stillbirth and complications in newborns. In 30–40% adults, it evolves towards potentially fatal complications after decades of silent progression[2]. Infections in the acute phase and, to a lesser extent the chronic one, can be treated with the aim of cure and interruption of transmission, or at least a reduction in the risk of morbidity[5].

Globally, the disease is largely under-diagnosed (an estimated 90% affected people are unaware of their infection and thus at risk of transmitting it and suffering complications[6]) and under-treated (less than 1% of affected individuals have access to treatment[7]). The World Health Organization (WHO) recommends that the diagnosis of chronic CD should rely on concordant results of at least two different serological tests based on different antigens[8]. Traditionally, conventional tests based on crude antigens/parasite lysate (enzyme-linked immunosorbent assay, ELISA; immunofluorescence test, IFAT; indirect hemagglutination test, IHA) are paired with non-conventional ones (mainly ELISAs) based on recombinant antigens[9]. Blood culture and polymerase chain reaction (PCR) are not considered sufficiently sensitive for the diagnosis during the chronic phase due to the intermittent and low-level peripheral parasitemia found throughout this period[10].

Rapid diagnostic tests (RDTs) are easy-to-use and less technically and time demanding than classical serological techniques. Remarkably, many of them can be performed on serum or with a very little volume of whole blood, and they can be stored on the shelf for longer than a year. Their large-scale use could contribute to increased access to diagnosis, better treatment coverage, and a reduction of disease transmission. Yet, despite having commonly been used for field surveys, RDTs are not recommended by the WHO[11–20].

The aim of this study is to review the accuracy of RDTs in field conditions for the diagnosis of chronic CD in populations at risk living in endemic and non-endemic countries.

## Methods

The protocol was registered with Prospero International prospective register of systematic reviews (record: CRD42016025990) on May 6^th^, 2016.

### Search strategy and selection criteria

We searched PubMed, SCOPUS, LILACS on 26^th^ November 2015 and up-dated the search on 01^st^ July 2017, without language or date limits. Original search strategy is available in Prospero (https://www.crd.york.ac.uk/prospero/display_record.php?RecordID=25990). Non-electronic sources have been also searched, like references listed in included studies or non-published data from expert in the field.

## Inclusion criteria

a) clinical studies with cohort recruitment (phase III studies) in field conditions; b) the presence of adequate reference tests (from now called "Reference Standard", RS) for the diagnosis of CD, namely a combination of two (or more) serological tests based on different techniques (either ELISA, IFAT or IHA) and antigens according to current WHO recommendations[8], or one or more high specificity test such as radioimmunoprecipitation analysis—RIPA or immunoblot or western blot, or the use of latent class analysis (LCA)-based reference standard; c) studies conducted on individuals (adults or children) with epidemiological risk of exposure to *T*. *cruzi* such as living in endemic area for at least one month, receiving blood transfusion in an endemic country or being born to a Latin American mother. We classified studies, on the basis of the sampling method, as being consecutive or non-consecutive. Case-control studies and those testing RDTs during the acute infection phase were excluded.

### Data collection

Two authors independently selected the studies, on the basis of the inclusion criteria. In case of discordant opinion, a third author was involved. Data were extracted from selected studies and risk of bias was assessed through the QUADAS-2 tool[21]. As possible sources of heterogeneity, we explored: geographical area, commercial brand of index test, type of RS, disease prevalence, study size, and risk of bias.

### Statistical analysis

The values of sensitivity and specificity were automatically computed in RevMan 2014 (Version 5.312). Individual study results were graphically expressed by plotting the estimates of sensitivity and specificity and their 95% confidence intervals (CIs) through both forest plots and receiver operating characteristics (ROC) space. We assessed heterogeneity by visual inspection of forest plots of sensitivity and specificity, and through visual examination of ROC plot of the raw data. Heterogeneity was further investigated using a bivariate random-effects model[22] to obtain estimates of the between-studies variation in sensitivity and specificity and the correlation between the two. The same bivariate model was used to assess the operating point sensitivity and specificity of the diagnostic tests under scrutiny, together with likelihood ratios and summary diagnostic odds ratio (DOR), taking both heterogeneity and threshold effect into account.

All analyses were performed using all articles first, then they were repeated splitting the studies into two main subgroups: studies conducted in endemic areas (continental LA), and studies in non-endemic areas (other continents). This was considered the primary analysis. Based on the results of included studies, we further conducted a secondary analysis on datasets evaluating the RDT most frequently used, i.e. Stat-Pak. All analyses were performed using Stata IC 13.0.

## Results

**Fig 1:**
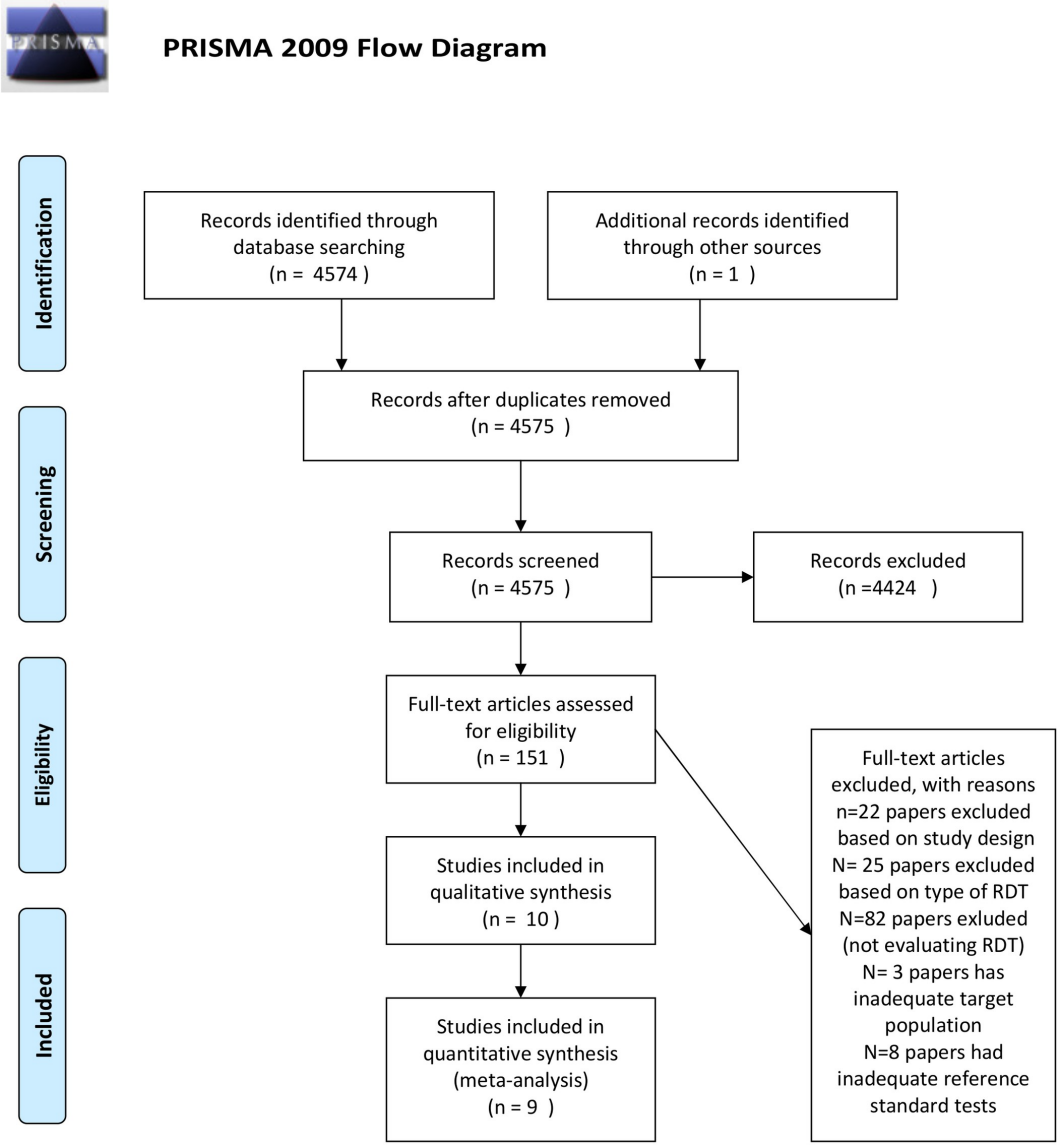
Study flow chart.

The electronic search identified 4574 records. The study flow is summarized in Fig 1.

Amongst the 151 articles included for full text evaluation, we excluded from the analysis studies on RDTs not based on immunochromatographic technique, because the 25 identified studies using other techniques were either evaluating old, out-of-market tests or were old studies for which we could not get access to the full text article[23–47]. Moreover, 22 papers were excluded on the base of the study design[48–69]. Eighty-two articles were excluded because they did not evaluate RDTs accuracy. Three papers were not eligible for inadequate target population (not chronic CD)[70–72], whereas 8 papers declared an inadequate reference standard for inclusion in the review[73–80].

“Eventually, 9 and 10 studies were included in the quantitative and qualitative analyses, respectively”. Two studies evaluated more than one single RDT, hence each dataset from those studies was considered separately[18,19]. One study[81] reported the results of a test conducted on a relatively small number of patients (114 children) that only had true negatives, providing no information on sensitivity. Thus, as we intended to model sensitivity and specificity jointly, we decided to exclude it from our analyses.

Finally, we included in the analysis 12 datasets, comprising a total of 6123 participants (from 101 to 1913 individuals tested in single studies). Six different RDTs were evaluated; Stat-Pak was assessed on more than half of the overall population included in the analysis (4 studies comprising 3347 participants, 53.3% of individuals included in our meta-analysis). Studies evaluating Stat-Pak were quite heterogeneous in terms of age-range of the selected population: one study included participants with more than 16 years of age; Bern et al. enrolled adults, Roddy et al. children and adolescents, and Eguez et al. individuals of all ages[11,18,19]. However, the participants resulted rather homogeneous in terms of origin, as three studies were conducted in Bolivia, and the study implemented in Europe comprised 47.4% of immigrants of Bolivian origin (474 of 999 participants)[12].

Among the other RDTs, one (Simple CHAGASWB, Operon) was evaluated by two studies with a total of 377 participants[15,20]. Both studies were conducted in Spain, and the reported prevalence of CD was 15.9% in the study by Navarro et al. (all ages, 76.4% of participants coming from Bolivia) and 5.9% in the work by Lopez-Chejade et al. (Latin American adults). Simple CHAGASWB showed a sensitivity/specificity of 88%/94.2% and 100%/96.8%, respectively, in Navarro et al. and Lopez-Chejade et al. studies.

Eguez and colleagues assessed the accuracy of a combination of two RDTs (Stat-Pak and InBios) against conventional tests (namely, IHA, lysate-antigen ELISA, and recombinant antigen ELISA)[19]: for the purpose of this review the study was split into two datasets, each one evaluating one RDT, while the combination of Stat-Pak and InBios was not included in the analysis.

Table 1 shows data and characteristics of each RDT and the reference standard for each study.

**Table 1:** Characteristics of studies.

References	RDT (index test)	Reference tests	Period of study	Country of implementation	Type and Number of participants	RDT Sensitivity/Specificity
Angheben 2017 [14]	Chagas Quick Test	ELISA para Chagas III, (BioChile, Chile) and Bio-Elisa Chagas, (Biokit, Spain)	2009–2015	Italy	Migrants from Latin America, all age, 640	83%/99%
Bern 2009_a [18]	InBios—Trypanosome detect	In-house IFAT, Chagatek ELISA (BioMerieux, Lab. Lemos, Argentina), and Chagatest ELISA Recombinante (Wiener lab., Argentina)	2006–2007	Bolivia	Bolivian pregnant women, 519	91%/100%
Bern 2009_b [18]	Stat-Pak		2006–2007	Bolivia	Bolivian pregnant women, 530	90%/100%
Brutus 2008 [17]	InBios—Trypanosome detect	IHA (Polychaco, Argentina) and Chagatest ELISA Recombinante (Wiener lab., Argentina)	2002–2004	Bolivia	Bolivian pregnant women, 460	93%/99%
Chappuis 2010 [12]	Stat-Pak	ELISA cruzi (bioMérieux Diagnostica, Brazil) and Bio-Elisa Chagas, (Biokit, Spain) + results of quality control of a reference lab in Brazil (performing other 4 serology tests)	2009	Switzerland	Migrants from Latin America, Adults, 999	96%/100%
Eguez 2017_a [19]	Stat-Pak	IHA (Polychaco, Argentina), Chagatest ELISA Recombinante (Wiener lab., Argentina), Chagatest ELISA Lisado (Wiener lab., Argentina)	2014	Bolivia	Bolivians from >1 years old up to 60 years old), 342	99%/100%
Eguez 2017_b [19]	InBios—CDP					90%/100%
Lopez-Chejade 2010 [15]	Simple Chagas WB	ELISA in house and BioELISA Chagas	Not declared	Spain	Migrants from Latin America, Adults, 148	100%/97%
Mendicino 2014 [13]	WL Check Chagas test	Chagastest ELISA, IHA, IFAT for discrepancies	Not declared	Argentina	Patients attending Primary Health Care Centers, 238	96%/100%
Navarro 2011 [20]	Simple Chagas WB	IFAT and ELISA (not specified)	2008–2009	Spain	Migrants from Latin America, all age, 276	88%/94%
Roddy 2008 [11]	Stat-Pak	Chagastest ELISA, Indirect hemagglutination test (HAI) (Polychaco, Argentina)	2007	Bolivia	Bolivians from >6 months to 17,9 years old, 1913	93%/99%
Shah 2014 [16]	InBios—CDP	Indirect hemagglutination test (HAI) (Polychaco, Argentina), IFAT, Chagatest ELISA Recombinante (Wiener lab., Argentina) or Chagatest ELISA Lisado (Wiener lab., Argentina)	2011–2012	Bolivia	Bolivians from >2 to 17 years old, 200	100%/99%

RDT = rapid diagnostic test; ELISA = Enzyme-linked immune assay; IHA = Indirect hemagglutination test; IFAT = Immunofluorescent antibody test

Antigens composition of the RDTs according to manufacturers:

“Chagas Quick Test” is based on *T*.*cruzi* specific antigens not better specified;

“InBios—Trypanosome detect” is based on a recombinant multiepitope fusion antigen: ITC8.2;

“Stat-Pak” is based on antigens B13, 1F8 and H49/JL7;

“InBios—CDP” is based on a recombinant multiepitope fusion antigen: ITC8.2;

“Simple Chagas WB” is based on a recombinant multiepitope protein: "Pep2-TcD-TcE-SAPA;

“WL Check Chagas test” is based on *T*.*cruzi* specific antigens not better specified.

Four studies were conducted in non-endemic areas (namely, Spain, Switzerland, and Italy)[12,14,15,20]; all studies conducted in LA were carried out in Bolivia, but one that was conducted in Argentina[13]. A couple of studies were conducted in a cohort of children while the others included either adults or individuals of all ages.

**Fig 2:**
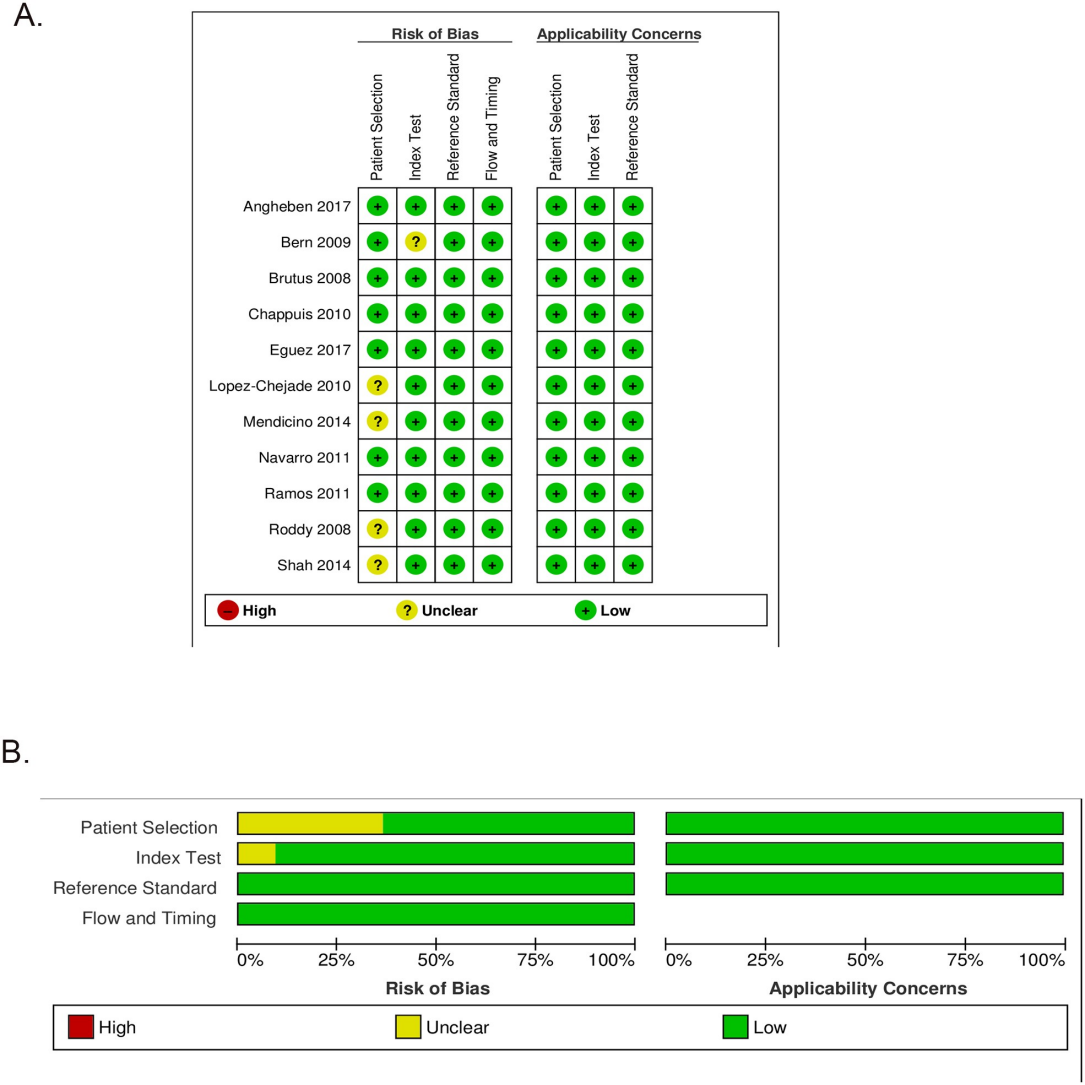
(a) Risk of bias and applicability concerns graph. (b) Risk of bias and applicability concerns summary.

The qualitative evaluation, in terms of rating for each study finally included in the analysis, and their overall methodological quality are shown in Fig 2a and 2b, respectively.

In general, the risk-of-bias and applicability concerns of all studies analyzed were considered low. Patient selection was assessed as “unclear” in four cases: the main reason was that the papers did not specify methods for the enrollment of patients (consecutive recruitment or random inclusion). Moreover, one study[18] did not clearly state if the results of the index tests were interpreted without knowledge of the results of the RS, hence the risk of bias in relation to the index test was assessed as unclear[18].

**Fig 3:**
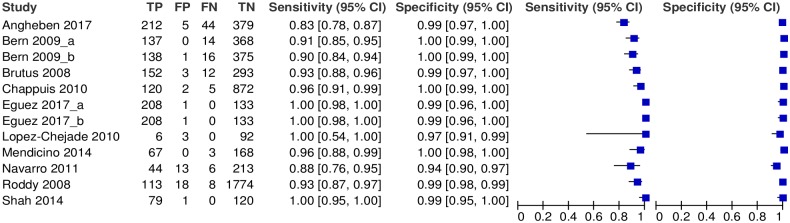
Forest plot displaying the accuracy of the RDTs by study Abbreviations: TP = true positives; FP = false positives; FN = false negatives; TN = true negatives.

Fig 3 shows the accuracy of the RDTs according to each dataset. Notably, heterogeneity among results of different studies was low, particularly in terms of specificity. Namely, the variance of the logit of the sensitivity resulted 1·82 (95% CI: 0·55 to 5·00), whereas the variance of the logit of specificity was 1·01 (95% CI: 0·29 to 3·41). The correlation between logit of sensitivity and logit of specificity resulted 0·34 (95% CI: -0·50 to 0·86). Globally, the accuracy of all RDTs resulted in: sensitivity = 96.6% (95% CI: 91.3–98.7%) and specificity = 99.3% (95% CI: 98.4–99.7%) (Table 2).

**Table 2:** Summary of findings of the review of immunochromatographic RDTs for the diagnosis of chronic CD in at risk populations.

Interpretative criteria: Endemicity / Stat-Pak	Effect (95% CI)	# of studies	Mean Prevalence^a^ (95% CI)	What do these results mean?
All areas	Sensitivity: 96.60% (91.3–98.7); Specificity: 99.27% (98.4–99.7)	12	30.33% (18.6 to 42.0)	Of the 30 out of 100 patients with CD, 1 will be missed by a single RDT (3.40% of 30). Of the other 67, not even 1 will have a false positive result of the RDT.
Endemic areas	Sensitivity: 98.07% (91.6–99.6); Specificity: 99.30% (98.3–99.7)	8	35.93% (20.7 to 51.1)	Of the 36 out of 100 patients with CD, not even 1 will be missed by a single RDT (1.93% of 33). Of the other 64, not even 1 will have a false positive result of the RDT.
Non-Endemic areas	Sensitivity: 89.77% (79.7–95.1); Specificity: 98.52% (95.0–99.5)	4	19.14% (4.3 to 42.6)	Of the 19 out of 100 patients with CD, 2 will be missed by a single RDT (10.23% of 19). Of the other 81, 1 will have a false positive result of the RDT.
Stat-Pak tests only	Sensitivity: 97.02% (87.6–99.3); Specificity: 99.44% (98.6–99.8)	4	26.37% (2.9 to 49.8)	Of the 26 out of 100 patients with CD, not even 1 will be missed by a single Stat-Pak test (2.98% of 26). Of the other 74, not even 1 will have a false positive result of the Stat-Pak test.

^a^ Estimates of true prevalences for each study were calculated as described by Rogan and Gladen (1978) [ref.]. CI: confidence interval; RDT: Rapid Diagnostic Test; CD: Chagas disease.

The RDTs showed better accuracy when used in endemic areas (Table 2): 98.1% and 99.3% respectively averaged sensitivity and specificity, whereas in non-endemic areas their sensitivity resulted lower: about 90%. The overall sensitivity of Stat-Pak was 97% (95% CI 87.6–99.3) and its specificity 99.4% (95% CI 98.6–99.8).

## Discussion

Globally, the sensitivity of the RDTs examined was good (higher than 95%) and the specificity was excellent (>99%), regardless their use in endemic or non-endemic regions. The sensitivity was basically higher in endemic (namely Bolivia) than in non-endemic areas. However, it must be noted that the latter data was obtained from four studies comprising 2063 individuals (around one third of the whole study population), with a lower proportion of Bolivians (35,9%, excluding Lopez-Chejade study, where the origin of migrants is not detailed) [12,14,15,20]. A previous study comparing 11 marked-available RDTs found out 8 tests which were considered valuable for clinical purpose (performances generally >90%). However, this was a case-control study based on selected serum samples, hence the accuracy of the tests could be overestimated [66].

In our work, among all RDTs Stat-Pak could be evaluated individually thanks to the high number of individuals tested. This test showed high accuracy, and its functionality with a little volume (10 μl) of whole blood further supports its use for screening purposes, as well as in field surveillance of the disease.

On the other hand, the number of studies (and participants included) addressing the other RDTs was too low to allow a separate meta-analysis. It must also be considered that *T*. *cruzi* population is characterized by a genetic polymorphism that might account at least in part for its variability in pathogenicity and transmission. Currently, seven genetic lineages or Discrete Typing Units (DTU) have been characterized, TcI-TcVI and Tcbat[82,83]. Their distribution varies geographically. Most studies included in this work concerned nationals of Bolivia or neighbouring countries (mainly the Gran Chaco area), where the the TcV prevails[83]. Different DTUs are prevalent in other regions (i.e. Mexico and Central America), with different antigenic features, tissue tropism, and pathogenicity profile. Therefore, our results cannot be automatically transferred to other CD epidemiological contexts. Moreover, all studies, both in endemic and non-endemic countries, concerned populations with high prevalence of *T*. *cruzi* infection. In populations with lower prevalence, our findings may not be entirely applicable.

On the other hand the Reference Standards, although based on different tests, were generally similar across selected studies: for all 12 datasets at least two paired tests were used (in two studies three tests were used and case definition was based on at least two concordant results, however)[18,19]: this permitted an accurate comparison between studies. We formally assessed risk of bias through the QUADAS-2 tool, and most of the selected studies received high scores, which further contribute to the robustness of our analysis. Finally, the assessment of variation in sensitivity and of the degree of correlation between sensitivity and specificity provided evidence of limited heterogeneity among studies. The utilization of statistical techniques that consider heterogeneity and threshold effect for the estimation of summary measures, such as the bivariate model suggested by Reitsma et al.[22], allowed the achievement of exhaustive and robust estimates.

### Applicability of findings

All studies included in our review were conducted under real-life conditions on populations at risk of having chronic CD. The practical implications of this statement are better summarized in Table 2. The use of a RDT would appear more appropriate for endemic than non-endemic areas, as in the latter a RDT-based screening would miss about 2 out of 19 infected subjects among 100 individuals tested. In contrast, in the endemic areas the proportion of infected subjects was higher (36 subjects with CD out of 100 tested), but less than one of the 36 would be missed by the RDT. On the other hand, in all contexts, the number of non-infected subjects erroneously found positive would be absolutely negligible, and the only significant consequence (in case a second, confirmatory test was not performed) would be to propose an unnecessary treatment. Considering only Chagas Stat-Pak test, the figures would be very similar, although in this case a comparison between endemic-non endemic areas was not possible.

While no RDT is sensitive enough to recommend its use for blood/organ donor screening, the technique appears to be sufficiently accurate for the screening of individuals at risk who could benefit from treatment. Considering that a strategy based on a single RDT would be much easier and cheaper to implement than the classical strategy based on two serological tests, it is very plausible to assume that the few cases missed (RDT false negatives) would be amply compensated by a larger population screened. This is especially valuable in rural areas of the endemic countries where access to diagnosis may be problematic. On the other hand, all positive individuals to a RDT should be submitted to a confirmatory test whenever possible, in order to avoid the side effects of an unnecessary treatment, as well as the stigma associated to the infection, for a false-positive subject. Alternatively, one of the studies included in this review[19] suggested the combined use of two RDTs as a strategy to increase the accuracy for screening purpose. The combination reached a near-perfect sensitivity (considering at least one positive out of two) and specificity (both positive).

### Conclusions

The accuracy of all RDTs under study can be considered sufficiently good to recommend their use in endemic settings, particularly in the Southern Cone of LA, in order to increase access to diagnosis. The Stat-Pak test can be recommended for use in screening surveys when the expected prevalence is moderately high or high, in the setting of Southern Cone or for migrants from that area in case of non-endemic countries[12]. On the other hand, the pooled sensitivity of all RDTs studied resulted too low to recommend them as stand-alone tests for detection of CD affected individuals in a non-endemic context, as a negative result cannot rule out a *T*. *cruzi* infection with reasonable certainty.

The WHO’s principle which states the need of diagnosis confirmation through another serologic test remains a prudent approach that should be followed at least for the confirmation of positive results.

Still, further studies conducting head-to-head comparisons of different available RDTs are needed, and it would be particularly important to extend these studies to the Andean countries, the Amazon basin, Central America and Mexico. Similarly, further studies will be required to ensure the applicability of RDTs in non-endemic settings. Robust evidence from studies of high quality is also needed to advocate adequate control policies and quality assurance in endemic countries, mainly in those with lower prevalence of the disease.

## Supporting information

S1 Checklist Study PRISMA-2009-checklist. (RTF)
