# Supplementary material for: Rapid immunochromatographic tests for the diagnosis of chronic Chagas disease in at-risk populations: A systematic review and meta-analysis
Source: PLoS Negl Trop Dis. 2019 May 31;13(5):e0007271. doi: 10.1371/journal.pntd.0007271 (PMC6561601; doi:10.1371/journal.pntd.0007271)
Supplement: S1 Checklist — (RTF) [file pntd.0007271.s001.rtf]

Section/topic 	#	Checklist item 	Reported on page # 	
TITLE 		
Title 	1	Identify the report as a systematic review, meta-analysis, or both. 	Title	
ABSTRACT 		
Structured summary 	2	Provide a structured summary including, as applicable: background; objectives; data sources; study eligibility criteria, participants, and interventions; study appraisal and synthesis methods; results; limitations; conclusions and implications of key findings; systematic review registration number. 	Abstract	
INTRODUCTION 		
Rationale 	3	Describe the rationale for the review in the context of what is already known. 	Introduction	
Objectives 	4	Provide an explicit statement of questions being addressed with reference to participants, interventions, comparisons, outcomes, and study design (PICOS). 	Introduction, last paragraph	
METHODS 		
Protocol and registration 	5	Indicate if a review protocol exists, if and where it can be accessed (e.g., Web address), and, if available, provide registration information including registration number. 	Methods, first paragraph	
Eligibility criteria 	6	Specify study characteristics (e.g., PICOS, length of follow-up) and report characteristics (e.g., years considered, language, publication status) used as criteria for eligibility, giving rationale. 	Methods, “Search strategy and selection criteria” paragraph
	
Information sources 	7	Describe all information sources (e.g., databases with dates of coverage, contact with study authors to identify additional studies) in the search and date last searched. 	Methods, “Search strategy and selection criteria” paragraph
	
Search 	8	Present full electronic search strategy for at least one database, including any limits used, such that it could be repeated. 	Methods, “Search strategy and selection criteria” paragraph	
Study selection 	9	State the process for selecting studies (i.e., screening, eligibility, included in systematic review, and, if applicable, included in the meta-analysis). 	Methods, “Data collection” paragraph	
Data collection process 	10	Describe method of data extraction from reports (e.g., piloted forms, independently, in duplicate) and any processes for obtaining and confirming data from investigators. 	Methods, “Data collection” paragraph	
Data items 	11	List and define all variables for which data were sought (e.g., PICOS, funding sources) and any assumptions and simplifications made. 	Methods, “Search strategy and selection criteria” and “Data collection”paragraphs	
Risk of bias in individual studies 	12	Describe methods used for assessing risk of bias of individual studies (including specification of whether this was done at the study or outcome level), and how this information is to be used in any data synthesis. 	Methods, “Data collection” paragraph	
Summary measures 	13	State the principal summary measures (e.g., risk ratio, difference in means). 	Methods,”statistical analysis” paragraph	
Synthesis of results 	14	Describe the methods of handling data and combining results of studies, if done, including measures of consistency (e.g., I2) for each meta-analysis. 	Methods,”statistical analysis” paragraph	

Page 1 of 2 
Section/topic 	#	Checklist item 	Reported on page # 	
Risk of bias across studies 	15	Specify any assessment of risk of bias that may affect the cumulative evidence (e.g., publication bias, selective reporting within studies). 	Methods, “Data collection” paragraph	
Additional analyses 	16	Describe methods of additional analyses (e.g., sensitivity or subgroup analyses, meta-regression), if done, indicating which were pre-specified. 	Methods,”statistical analysis” paragraph	
RESULTS 		
Study selection 	17	Give numbers of studies screened, assessed for eligibility, and included in the review, with reasons for exclusions at each stage, ideally with a flow diagram. 	Figure 1	
Study characteristics 	18	For each study, present characteristics for which data were extracted (e.g., study size, PICOS, follow-up period) and provide the citations. 	Results, first 4 paragraphs and Table 1	
Risk of bias within studies 	19	Present data on risk of bias of each study and, if available, any outcome level assessment (see item 12). 	Figures 2a and b and Results, paragraph 5	
Results of individual studies 	20	For all outcomes considered (benefits or harms), present, for each study: (a) simple summary data for each intervention group (b) effect estimates and confidence intervals, ideally with a forest plot. 	Figure 3, paragraph 6 of Results	
Synthesis of results 	21	Present results of each meta-analysis done, including confidence intervals and measures of consistency. 	Table 2 and Resuls, last paragraph	
Risk of bias across studies 	22	Present results of any assessment of risk of bias across studies (see Item 15). 	Figures 2a and b and Results, paragraph 5	
Additional analysis 	23	Give results of additional analyses, if done (e.g., sensitivity or subgroup analyses, meta-regression [see Item 16]). 	Table 2 and Resuls, last paragraph	
DISCUSSION 		
Summary of evidence 	24	Summarize the main findings including the strength of evidence for each main outcome; consider their relevance to key groups (e.g., healthcare providers, users, and policy makers). 	Discussion, “Applicability of findings” paragraph	
Limitations 	25	Discuss limitations at study and outcome level (e.g., risk of bias), and at review-level (e.g., incomplete retrieval of identified research, reporting bias). 	Discussion, second paragraph	
Conclusions 	26	Provide a general interpretation of the results in the context of other evidence, and implications for future research. 	Discussion, “conclusions” paragraph	
FUNDING 		
Funding 	27	Describe sources of funding for the systematic review and other support (e.g., supply of data); role of funders for the systematic review. 	NA	

From:  Moher D, Liberati A, Tetzlaff J, Altman DG, The PRISMA Group (2009). Preferred Reporting Items for Systematic Reviews and Meta-Analyses: The PRISMA Statement. PLoS Med 6(6): e1000097. doi:10.1371/journal.pmed1000097 
For more information, visit: www.prisma-statement.org. 
Page 2 of 2 
